# *let-7* microRNAs: Their Role in Cerebral and Cardiovascular Diseases, Inflammation, Cancer, and Their Regulation

**DOI:** 10.3390/biomedicines9060606

**Published:** 2021-05-26

**Authors:** David L. Bernstein, Xinpei Jiang, Slava Rom

**Affiliations:** 1 Department of Pathology and Laboratory Medicine, Lewis Katz School of Medicine, Temple University, Philadelphia, PA 19140, USA; david.bernstein@temple.edu (D.L.B.); xinpei@temple.edu (X.J.); 2 Center for Substance Abuse Research, Lewis Katz School of Medicine, Temple University, Philadelphia, PA 19140, USA

**Keywords:** microRNAs, let-7, stroke, cardiovascular, inflammation

## Abstract

The *let-7* family is among the first microRNAs found. Recent investigations have indicated that it is highly expressed in many systems, including cerebral and cardiovascular systems. Numerous studies have implicated the aberrant expression of *let-7* members in cardiovascular diseases, such as stroke, myocardial infarction (MI), cardiac fibrosis, and atherosclerosis as well as in the inflammation related to these diseases. Furthermore, the *let-7* microRNAs are involved in development and differentiation of embryonic stem cells in the cardiovascular system. Numerous genes have been identified as target genes of *let-7*, as well as a number of the *let-7*’ regulators. Further studies are necessary to identify the gene targets and signaling pathways of *let-7* in cardiovascular diseases and inflammatory processes. The bulk of the *let-7*’ regulatory proteins are well studied in development, proliferation, differentiation, and cancer, but their roles in inflammation, cardiovascular diseases, and/or stroke are not well understood. Further knowledge on the regulation of *let-7* is crucial for therapeutic advances. This review focuses on research progress regarding the roles of *let-7* and their regulation in cerebral and cardiovascular diseases and associated inflammation.

## 1. The let-7 Family and Inflammation

**Figure 1:**
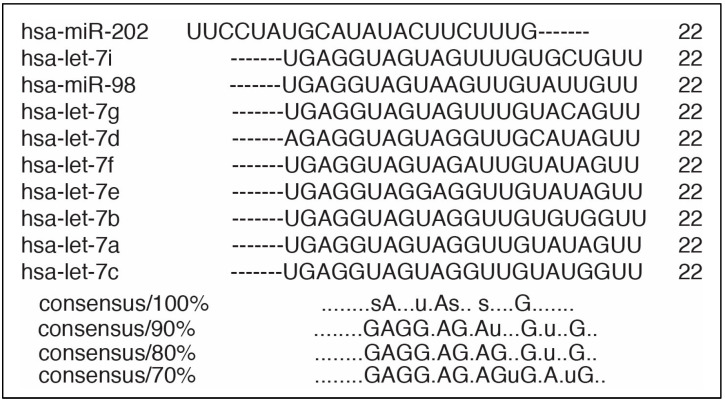
Sequence alignment of the *let-7* microRNAs. Performed with ClustalW tool (https://www.genome.jp/tools-bin/clustalw (accessed on 2 April 2021).

The *let-7* family of microRNAs is one of the earliest originally discovered microRNAs. When several isoforms were identified in *C. elegans* in 2000 [1], the miR was named *lethal-7* (*let-7*) because its knockout was lethal during development [2]. The discovery of *let-7*, along with lin-4 [3], opened much of the current field of miR research. To date, 12 genetic loci have been identified as origination sites of *let-7* in humans [3], while mice have 3 [4] and drosophila have 1 [5]. In humans and mice, 10 of the *let-7* microRNAs (miRs) are present (*let-7a, b, c, d, e, f, g, i*, and miR-98 and miR-202) [3]. However, in spite of the multiple sites of origin, all *let-7* miRs begin as pre-pro *let-7* transcripts and are then processed through the Drosha pathway [6]. Throughout miRNA biogenesis, after Dicer cleavage, one of the strands is loaded into an RNA-induced silencing complex (RISC) as mature miR. The other strand, which is labelled as the “star strand”, is typically degraded [7,8], though for certain miRs, both strands are preserved and are loaded into RISC as mature forms. In such an instance, the mature miR is called, for example, with *let-7a* miR, as *let-7a-5p* and *let-7a-3p* or *let-7a* and let-7a*, respectively. Despite being present in many different genetic regions, all final *let-7* miRs are similar in length, differing by only 0–3 nucleotides (Figure 1), although their functions differ significantly in protein translation and physiological function.

As one of the first miR families discovered, much has been investigated about the multiple roles that the *let-7* family plays within the body. Numerous studies have linked *let-7* miRs to many processes, from cell proliferation [9,10] and bone remodeling [11] to cardiac output [12]. However, across many tissues and conditions, miRs from the *let-7* family appear to be particularly involved in the signals involved in the growth and stress responses of many types of cells, particularly after extracellular insults. Consequently, many *let-7* miRs confer significant impact on the regulation of inflammatory processes, including within the central nervous system (CNS). To date, all nine constituent members of the *let-7* family have been linked to regulation of vascular function and neurological outcomes. For example, upregulation of *let-7a* and *let-7c* has been associated with protection from ischemia [13], improved responses to spinal cord ischemia/reperfusion [14], and protection from neuroinflammation [15]. In particular, *let-7a* can induce a significant effect on vascular function, as it appears to regulate post-stroke angiogenesis through a transforming growth factor beta 3 (TGF-β3)-dependent mechanism [16]. By contrast, post-insult expression of *let-7b*, which differs from *let-7a* by a single nucleotide, is associated with greater vascular damage, including in ischemic heart tissue following myocardial infarct (MI) [17]. Furthermore, its expression remains elevated for weeks after multiple forms of ischemic injury, including large and small vessel stroke and cardiac embolism [18]. In fact, the elevated presence of *let-7b* is considered strongly predictive of poor outcome following ischemic stroke [19,20]. Such differences underscore the complexity of *let-7*’s role in modulating vascular responses following inflammation and underly some of the difficulty in correcting *let-7* expression following insult. 

Two members of the *let-7* family, let-7g* and *miR-98*, were shown to be critical for modulating the vascular response to hypoxia. Both miRs were significantly downregulated during hypoxic events in vitro and in whole animal models. More significantly, restoration of endogenous levels of let-7g* and *miR-98* expression appears to prevent a significant degree of damage from ischemia and stroke and to improve functional recovery [21,22,23,24]. The strong neuroprotective effect of increased *miR-98* and let-7g* expression was observed even when such treatments are given 24 h after ischemia/reperfusion [21,23,25]. Upon restoration of endogenous *miR-98* or let-7g* expression, researchers have noted preservation of blood–brain barrier (BBB) integrity, reduction of pro-inflammatory cytokine release, prevention of immune cell infiltration into the infarcted region, and an overall decrease in the size of the ischemic penumbra, leading to improved behavioral outcomes [21,23,25].

In addition to *mir-98* and let-7g*, other members of the *let-7* family have been shown to produce various neuroprotective or neuroinflammatory roles following various CNS insults. *let-7i* expression is somewhat correlated with the impact of reperfusion injury, and its expression is strongly correlated with preservation and recovery of post-stroke function [26]. This may be due to its role in regulating leukocyte attachment and recruitment to the brain endothelium [27], along with its importance in other mechanisms of endothelial self-repair [28]. *let-7c* is critical for mediating both the recruitment of immune cells to ischemic tissue [19] as well as the activation of multiple repair pathways within the endothelium [20]. While the above-mentioned *let-7* miRs demonstrate anti-inflammatory and protective characteristics, *let-7e* has been shown to be an early proinflammatory marker of hypoxic damage, and it may further propagate damage [29,30].

The anti- and pro- inflammatory pathways regulated by *let-7* miRs are not confined to the CNS. *let-7i* has been associated with wound repair across many cell types, due in part to interactions with progesterone [31]. *let-7c* has been shown to be associated with regulation of dental inflammation through extracellular matrix (ECM)-specific mechanisms [32]. *let-7d* is involved in recovery from hypoxia in cardiac tissue and offers therapeutic potential for treating the aftermath of MI [33] by stimulating proliferation and activating survival pathways in cardiac cells. *let-7g* shows similarly strong potential for stimulating cell repair within ischemic heart and vascular tissue [34] and has been shown to promote angiogenesis through activation of vascular endothelial growth factor (VEGF)-mediated signaling following insult [34,35]. 

**Figure 2:**
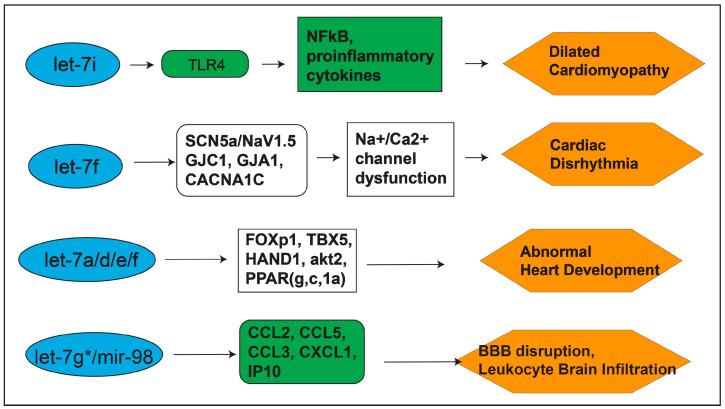
Targets of the *let-7* miRs in cerebral and cardiovascular disease conditions. Green boxes show confirmed targets, while white boxes show bioinformatic prediction [13,21,23,24,25,26,36,37].

Taken together, the *let-7* family appears critical for the progression of inflammation. However, its individual members can have significant and sometimes contradictory impacts on such processes. One possible explanation is the timeline in which expression occurs. Certain *let-7* family members such as *let-7a* and *let-7e* are associated with early inflammatory and pre-apoptotic pathways, while others such as *let-7i* and *let-7f* are involved in later phases of transcription and downstream elements of apoptosis (Figure 2). In the following section, we further expand on the particular regulatory elements of *let-7* miRs and why the timeline of expression of these miRs and subsequent impact on cytokine and chemokine release appear to have such high variability.

1.1. let-7 miRNAs, Cell Division, and Vascular Function

*let-7* is critical for prenatal development. It appears soon after fertilization, where it is responsible for mediating blastocyst attachment to uterine spiral arteries. Even from the beginning, *let-7* miRs work as clipping signals, minimizing the proliferation of non-adherent cells through suppression of transcription factors [2], thereby allowing only attached cells to grow. For this reason, *let-7* was named *“lethal-7”,* because its absence leads to uncontrolled cellular proliferation, preventing development into a viable embryo. This silencing-type role continues throughout later stages of embryonic development [36]. After the first trimester, *let-7* miRs are critical for the differentiation of different organs by arresting the proliferation of non-needed cells, functioning as a “stop” sign in many tissues, including lung [37], brain [36], heart, and vascular tissue [38]. In particular, *let-7* provides critical control of the length of the DNA replication phase in neural stem cells, thereby regulating the growth of the CNS from the first trimester through birth [39,40,41]. This critical regulation of cellular differentiation appears to be conserved across species; *let-7* is also necessary for differentiation in rodents [41] and invertebrates [5].

*let-7’*s role in the vascular system begins from day 1, as it is subject to regulation by chemokines secreted by endothelial cells (EC) within the uterus [2]. *let-7* expression is critical for maintaining the integrity of endothelial cells, and its normal expression is considered critical for maintenance of the blood–brain barrier in the face of ischemic disease [26]. These processes are not confined to periods of stress; *let-7* is also critical for maintenance of endothelial cell walls. In normal functioning, *let-7* is responsible for transducing fibroblast growth factor (FGF) signaling into changes to TGF-β within endothelial cells, thereby limiting proliferation [42,43] in non-damaged blood vessels. *let-7g* has been shown to reduce EC inflammation and monocyte adhesion [21,23,24,44], diminish EC senescence, and play a role in controlling arterial stiffness and aging [45,46]. The strong effect of *let-7* on vascular health and angiogenesis underscores its role in recovery from stroke and other vascular diseases processes, such as myocardial infarction [12,36]. However, *let-7*’s critical role in regulating EC division also makes the miR family a critical regulator of disorders of cell proliferation, such as cancer.

1.2. let-7’s Role in Cancer and Angiogenesis

In virtually all forms of cancer, continual tumor growth requires altered and abnormal angiogenesis [47]. Without additional vascular collaterals, tumor masses are unable to grow to larger than 1 mm in size [47]. Hence, as a critical regulator of angiogenesis, *let-7* is one of the most important elements in controlling the progression of cancer. Normal physiological levels of *let-7* effectively suppress abnormal angiogenesis and prevent tumor growth [48]. Conversely, suppression of *let-7* expression is a hallmark of most forms of cancer [49]. Moreover, tumors with lower tissue levels of *let-7* proliferate significantly faster than those with higher *let-7* expression [48,50]. Consequently, lower levels of *let-7* are associated with more aggressive growth and poor prognosis in cancer patients [51]. Perhaps most critically, restoration of *let-7* has been shown to have strong anticancer properties. This has led to it being classified as a tumor suppressor [52], as well as a promising target for future cancer therapies [53].

The inverse relationship between tissue expression of *let-7* and cell growth can be attributed in large part to actions on the vasculature. First, in patients with leukemia and lymphoma, *let-7* expression correlated inversely with the spread of the disease [54]. It has also been shown that reductions in *let-7* lead to more abnormal angiogenesis [55] and weaken vascular wall integrity [1]. Such effects may be combinatory and lead to greater inflammation, particularly IFNγ-mediated increases in cell aggregation [56], which can further exacerbate oxidative stress in the area and promote abnormal growth. 

*let-7* is considered a tumor suppressor gene, due in large part to its normal physiological role in arresting development. In many tissues, including vasculature, lung, and liver, proliferation is arrested by the presence of *let-7* miRNA. Currently, the *let-7* miRs have been associated with cancer. Part of this relationship is physical; *let-7* binds to coding regions and untranslated regions (UTRs) of genes critical for DNA replication such as programmed cell death ligand 1 (PD-L1) [53] and high mobility group AT-hook 2 (HMGA2) [57], as well as apoptotic genes such as caspase 3 [58], B-cell CLL/lymphoma (BCL) [59,60], and caspase 8 [61].

Recently, researchers have determined the bidirectional nature of *let-7* and cancer. miR-98 overexpression has been shown to reduce proliferation in many cells [62]. Its effect is particularly strong in endothelial cells, accounting for most forms of proliferative control [63,64]. It appears to regulate endothelial cell growth through multiple mechanisms, although a critical pathway of control is propagated through interactions with VEGF/Argonaute RISC component (AGO) pathway [36,65,66]. On the list of *let-7* targets are genes controlling cell signaling and cell cycle as well as differentiation. In some cases, *let-7s* are labelled as tumor suppressors because they reduce cancer aggressiveness. Nevertheless, in sporadic conditions, *let-7* acts as an oncogene, accelerating cancer migration, invasion, and chemoresistance due to expression of genes associated with progression and metastasis. For these reasons, *let-7s* might be considered as potential diagnostic and prognostic markers and therapeutic targets for cancer treatment [67].

## 2. Regulation of *let-7* Expression

*let-7* miRs are evolutionarily conserved across species and play essential roles in many biological processes due to their pluripotency, such as in differentiation, growth, proliferation, self-renewal, development, and diseases. Due to the broad effects of *let-7* and any dysregulation leading to disease physiology, it is necessary to tightly control *let-7* expression. Therefore, it is critical to recognize what, where, and how *let-7* expression is regulated throughout its maturation process. *let-7*, as with any other miR, can be regulated transcriptionally and post-transcriptionally throughout the maturation process. miR (*let-7*) biogenesis and maturation are largely dependent on Drosha in the nucleus for primary microRNA (pri-miRNA), Dicer for precursor microRNA (pre-mRNA), and RISC for mature miRNA in the cytoplasm. Due to the complexity of miRNA biogenesis, its expression level can be regulated at different steps. miRNA can be regulated at transcription or post-transcription. Here, we discuss some known positive and negative protein regulators of *let-7* expression at different stages (Table 1).

### 2.1. Negative Transcriptional Regulation of let-7s 

*let-7* is one of the first miRNAs to be discovered, but its transcriptional regulation is not fully understood. It has been reported that DAF-12 nuclear hormone receptor and *let-7s* have a bimodal feedback loop at the transcription level in a ligand dependent manner. Unliganded DAF-12 inhibits the transcription of *let-7* in worms through a co-repressor, DIN-1 [68,69]. DAF-12 cannot bind directly to the endogenous ligands; the ligands bind DIN-1 and modulate the activity of DAF-12. With favorable environmental and developmental cues, the ligand binds the DIN-1/DAF-12 complex, and DAF-12 is able to directly activate *let-7* transcription [68,69]. Another interesting target that shares a bimodal feedback circuit with *let-7s* is MYC. Some studies have reported that MYC binds to the conserved promoter upstream of *let-7a-1/let-7f-1/let-7d* and *let-7g* polycistronic clusters in the *pri-let-7s* and suppresses its transcription [70,71]. Interestingly, DAF-12 and MYC 3′-UTRs contain *let-7* complementary sites that are targets of *let-7* [135,136]. In addition, LIN42, a period protein homolog, has been shown to regulate a wide variety of miRNAs through transcriptional repression of *let-7* family pri-miRNA production in worms [72]. LIN42 suppresses *let-7* phenotypes through transcriptional repression at the pri-let-7s promoter region as LIN42 protein level increases [1,72,73]. *let-7* has been also shown to have a complementary sequence to the 3′-UTR regions of many genes, such as, *lin41, lin28, lin42,* and *daf-12* [1]. In mammals, these proteins are represented by TRIM71, a LIN28 homolog, period circadian regulator, PCR, and nuclear hormone receptor, NHR [137,138], respectively. Bimodal regulation is prevalent in *let-7* regulation; it is essential to find more targets sharing a bimodal regulation loop with *let-7s* to further our understanding of the complexity of *let-7* regulation and biogenesis in vivo.

#### 2.1.1. LIN28-Dependent and -Independent Regulation of *let-7s* Biogenesis

LIN28s are considered as the master regulators of *let7s*. LIN28A and LIN28B paralogs are RNA binding proteins [74,139] and have direct roles in modulating *let-7* miRNAs. LIN28A and LIN28B can post-transcriptionally suppress both *pri-let-7s* and *pre-let-7s* biogenesis and maturation via both 3′ terminal uridylyl transferase (TUTase)-dependent (LIN28A) and -independent pathways (LIN28B) by inhibiting Drosha and Dicer activities [74,75,76,81]. LIN28A and LIN28B bind to both *pri-let-7* and *pre-let-7;* however, they work independently and distinctively. LIN28A is mainly in the cytoplasm; it recruits TUTases4/7 to oligo-uridylate *pre-let-7s* at its 3′ end. Uridylated *pre-let-7s* cannot undergo Dicer processing, which marks these *pre-let-7s* for degradation [74,77,78,79,80]. When LIN28A is absent, *pre-let7s* processing is upregulated, resulting in more mature *let-7s* [75]. This increase in *let-7s* is regulated by TUTases2/4/7; these proteins mono-uridylate group II *pre-let-7s*, except *pre-let-7a-2, 7c*, and *7e*, and enhance Dicer processing, resulting in increased *let-7s* [82]. Interestingly, in the cytoplasm LIN28A can selectively recruit TUTase4 to a subset of *pre-let-7s* to mediate uridylation processing and suppress pre-let-7 Dicer processing [77,78,80]. Oddly, LIN28B blocks *let-7* miRNA biogenesis via TUTase-independent pathways [79]. LIN28B is mainly located in the nucleus and sequesters *pri-let-7s* into the nucleolus and prevents Drosha/DGCR8-mediated *pri-let-7* processing [75,76,79]. Despite the similarities between the LIN28 paralogs, they work by discrete pathways at multiple steps and negatively regulate nearly all of *let-7* biogenesis. Due to the complexity of the LIN28/let-7 axis and context-dependent regulation of let-7s, it is necessary to further understand what other factors can modulate LIN28/let-7 axis. Several of the factors modulating *let-7s* biogenesis through the LIN28/Let-7 axis are discussed below.

#### 2.1.2. LIN28-Dependent Regulation

Fragile histidine triad diadenosine triphosphotase (FHIT) was found to induce LIN28B protein expression, leading to the suppression of *let-7s* [82]. Chae and colleagues showed that FHIT expression correlated inversely with *let-7* miRs, and FHIT apparently mediates the negative feedback initiated by LIN28/Let7 at the *pri-miRNA* level in the nucleus [84]. An additional LIN28B-regulating protein is mucin 1 (Muc1), a heterodimeric protein that is subsequently autocleaved to Muc1-N and Muc1-C [85]; the later translocates to the nucleus and interacts with transcription factor NF-κB p65 [87]. Kufe’s group demonstrated that Muc1-C activates LIN28B in an NF-κB-dependent manner and suppresses *let-7 biogenesis* [86]. Musashi1 (MSI1) protein either works in conjunction or compensates LIN28 to post-transcriptionally negatively regulate *miR-98, let-7b*, and *let-7g* biogenesis via Drosha processing [88]. Sjögren syndrome antigen B (SSB) protein has been shown to bind to the UUUOH element located in the 3′ end of LIN28B RNA transcripts [90,91] and subsequently to enhance LIN28B’ protein levels [89]. Whereas SSB’ silencing decreased LIN28B level, it successively resulted in an increase of mature *let-7s* (*7a, 7b, 7c, 7d, 7e, 7f, 7g,* and *7i*) through released inhibition of *pri-let-7* processing [89]. TRIM25 protein is an E3 ligase that binds to *pre-let-7s* conserved terminal loop and activates LIN28A/TUTase4-mediated uridylation [92]. It has been reported that TRIM25 is a cofactor for LIN28A/TUTase4-mediated uridylation and functions in cis to provide additional specificity and regulation of LIN28A in suppressing the maturation of pre-let-7s [77,78,92]. YAP, yes-associated protein, is a transcriptional coactivator that plays important roles in various cellular processes. YAP is downstream of the Hippo signaling pathway and has been reported to regulate miRNA biogenesis in a cell-contact-dependent manner [99,100]. At low cell density, unphosphorylated YAP translocates into the nucleus and sequesters p72, a DEAD-box helicase 17 (DDX17), which is an essential component of the miRNA processing machinery, Drosha/DGCR8, resulting in downregulation of *let-7* (*7a* and *7b*) [99,100]. When cell density and cell-to-cell contact increases, phosphorylated YAP remains in the cytoplasm and is unable to sequester DDX17; consequently, the later binds to Drosha/DGCR8 complex and increases the *pri-let-7s* processing [99,100]. YAP’s nuclear-cytoplasmic dynamics provides additional regulatory control to the LIN28/let-7 axis through a novel cell-contact-dependent miRNA biogenesis.

#### 2.1.3. LIN28-Independent Regulation

Heterogenous nuclear ribonucleoprotein A1 (HnRNPA1) has been shown to bind Drosha complex [109]. HnRNPA1 also binds the conserved terminal loop of *pri-let-7a-1* and inhibits its processing by Drosha and DGCR8 complex [108,109]. The binding of HnRNPA1 with the *pri-let7a* alters the pri-miRNA structure and inhibits Drosha processing [109,110]. HnRNPA1 depletion increases *pri-let-7a-1* processing, whereas ectopic expression of hnRNPA1 decreases *let-7a* [106]. Death receptor tumor necrosis factor-related apoptosis-inducing ligand (TRAIL)-R2 associates with p68 RNA helicase (DDX5), nuclear factor 90/45 (NF90/NF45), and hnRNPA1; together this complex is involved in RNA processing and gene regulation [117]. These binding partners of TRAIL-R2 have been shown to be involved in *let-7s* maturation and biogenesis [118,119]. Knockdown of either TRAIL-R2 or NF90/NF45 results in enhanced processing of *pri-let-7s* by the Drosha/DGCR8 complex and significant intensification of levels of different mature *let-7s* (*7a, 7b, 7c, 7d, 7e,* and *7g*) [117,120]. Several studies have reported that HnRNPA1’ binding to *let-7a* interferes with the binding of the KH-type splicing regulatory protein (KSRP), known to promote *let-7a* biogenesis [111,112]. HnRNPA1 and KSRP compete for *pri-let7* binding sties and reversibly regulate *let-7* biogenesis in vivo [109]. This antagonizing regulation of hnRNPA1 and KSRP adds an additional layer to *let-7* biogenesis and adds additional complexity to its homeostatic regulation that requires further investigation, especially under pathophysiological conditions, such as neuroinflammation, cardiovascular diseases, and stroke. STAUFEN1 protein has been shown to negatively modulate *let-7s* by binding to the *pri-let-7s* 3′-UTR and altering their structure and integrity [129]. The immune regulator, monocyte chemoattractant protein 1-induced protein 1 ribonuclease (MCPIP1), suppresses *pre-let-7s* miRNA biogenesis by inhibiting Dicer processing [132]. MCPIP1 also has an oligomerization domain for *pre-let-7g* recognition leading to its degradation [131]. 

### 2.2. Positive Regulation of let-7s Biogenesis

#### 2.2.1. LIN28-Dependent Positive Regulation

Due to the complexity of LIN28/let-7 regulation, many layers of regulation are necessary. One study revealed that TRIM71, an E3 ubiquitin ligase, negatively modulates LIN28B through polyubiquitination, leading to the upregulation of mature *let-a* and *pre-let-7s* post-transcriptionally [93]. TRIM71 levels are also dependent on LIN28 expression; a decrease in LIN28 will reduce TRIM71 expression [94]. Additionally, TRIM71 binds to the catalytically active Ago2 protein using its NHL domain, inducing the degradation of Ago2 by interfering with mature *let-7s* [94,95,96]. This adds a new layer of regulatory complexity to *let-7* biogenesis and maturation post-transcriptionally. Tristetraprolin (TTP) is an AU-rich pentamer element (ARE)-binding protein that has been reported to downregulate LIN28A via binding to LIN28A’ AREs in its 3′-UTR, resulting in subsequent degradation and promotion of *let-7s* maturation [97,98]. It is also interesting that AREs are often located in the 3′-UTR of various mRNA of cytokine mRNAs [98]. TTP presumably plays an important role in regulating inflammatory responses as well by directly binding to ARE-containing transcripts and downregulating these inflammatory response transcripts [140,141].

#### 2.2.2. LIN28-Independent Positive Regulation

Adenosine deaminases acting on RNAs (ADARs) have been reported to convert adenosine residues to inosine residues in pri-miRNAs, pre-miRNAs, and mature miRNAs and to modify their structures, functions, stability, and biogenesis [101,102,142]. Loss of ADAR1 was found to significantly downregulate *let-7a, 7b, 7d*, and *7e* expression through Drosha- and Dicer-mediated processing [103]. ADAR1 modulates the expression of *pri-let-7-Complex* (*let-7c*) locus through a single A-to-I change at the six residues of pri-miR polycistronic transcript, leading to enhanced miRNA processing by Drosha cleavage [101]. ADAR1 mediates the differential expression of many polycistronic miRNA clusters through direct binding to Drosha/DGCR8 or Dicer complexes, such as, *pri-*/*pre-let7-a-1*, *let-7a-2*, *let-7a-3*, *pri-/pre-let-7d*, and *pri-let-7f* [102,103,104,105,106]. Tumor suppressor breast cancer 1 (BRCA1) directly promotes the processing of pri-*let-7a* [121]. BRCA1 increases the expression of both primary transcripts and mature *let-7a*. BRCA1 was shown to directly interact with DDX5 and the Drosha complex, and studies found that BRCA1 associates with SMAD3, p53, and DEAH-box RNA helicase (DHX9) [121,122]. BRCA1 can directly bind to primary transcripts’ stem root via a DNA-binding domain and can regulate *let-7a* biogenesis via the Drosha/DGCR8 complex and SMAD3/p53/DHX9 [121]. It also has been reported that SMAD3 and p53 are involved in *let-7a* maturation [125,143] and interact with BRCA1–Drosha complex [126]. SMAD3, p53, and DHX9 interactions with BRCA1 likely strengthen and stabilize BRCA1-induced Drosha processing activity. Human nuclear interacting protein 1 (SNIP1) is an RNA-binding protein that interacts with Drosha complex and has been reported to function in TGF-β and NF-κB signaling pathways. SNIPP1’ downregulation resulted in *let-7i* reduction [127], confirming its positive regulation in *let-7* biogenesis. Another protein associated with Drosha/DGCR8 complex is synaptotagmin-binding cytoplasmic RNA-interacting protein (SYNCRIP), which was shown to bind to the conserved terminal loop within *pri-let-7* [144]. Silencing SYNCRIP reduces mature *let-7a* level, while overexpressing SYNCRIP promotes *let-7a* [144]. Depletion of the BCDIN3D, a member of the Bin3 family, revealed strong downregulation of a number of mature *let-7s* (*7b, 7d, 7e, 7f, 7g, 7i and miR-98*) [130]. BCDIN3D has been reported to interact with Dicer in an RNase A-dependent manner and facilitates Dicer processing [131]. Additionally, BCDIN3D has been shown to directly interact with *pre-let-7s* and methylate them in vitro with great specificity, leading to enhanced Dicer processing [130]. RBM3, a cold-inducible, developmentally regulated RNA-binding protein regulates *let-7* biogenesis [133,134]. RBM3 level has been shown to directly correlate with miRNA generation and vice versa [133]. Pilotte and colleagues have shown that changes in *pre-let7-a*, *pre-let-7g*, and *pre-let-7i* are affected by the presence of RBM3 [133]. RBM3 directly binds these pre-*let-7s* and enhances these precursors’ association with active Dicer complexes [133]. RBM3’ ability to directly bind to pre-miRNAs and regulate subsequent Dicer processing under hypothermia makes it an interesting target for modifying miR expression in temperature-sensitive processes. Another *let-7* biogenesis-involved protein is a TAR DNA-binding protein 43 (TDP-43). TDP043 belongs to the hnRNP family and has been shown to play a major role in many cellular processes [114,115]. Since hnRNPA1 has been described as associating with Drosha [107,113], likewise TDP-43 is a Drosha-associated protein [115,116] and is reported to downregulate *let-7b* [115]. *Pri-let-7b* binds directly to TDP-43 in different positions within the miRNA and/or the hairpin [115]. When TDP-43 is depleted, *let-7b* is downregulated [115]; this shows that TDP-43 plays a positive role in *let-7b* biogenesis. Intriguingly, another study did not find that depletion of TDP-43 lowered *let-7b* level [116]. These contradictory results remind us of the complexity of *let-7* biogenesis in a context-dependent manner, and further investigations are needed to discover TDP-43’s role in regulating *let-7* biogenesis.

## 3. *let-7’*s Protein Regulators and Their Role in Stroke and Other Cardiovascular Disease-Related Inflammation 

*let-7* is involved in many cellular processes, immunity, and protective functions. Regulators of *let-7* are crucial for therapeutic advances. The majority of these regulatory proteins are well studied in development, proliferation, differentiation, and cancer, but their roles in inflammation, cardiovascular diseases, and/or stroke are not well studied. Only a few of these regulatory proteins have been described as having a link with cardiovascular diseases and stroke outcomes. hnRNPA1 has recently been reported to interact with β-arrestin1 to upregulate a miRNA processing in the heart [145]. KSRP has been shown in vitro to regulate inflammatory responses [112] through controlling inflammatory mediators, such as TNFα, IL-1β, IFNα, and IFNβ expressions [146]. Similar to TTP, KSRP is involved in direct and indirect control of cytokine synthesis and degradation, potentially through miRNA regulation [146,147]. Upregulated SMAD3/TGF-β signaling has been reported to significantly increase cell survival and exhibit neuroprotective effects after cerebral ischemic stroke [148]. Cardiomyocyte apoptosis is considered a significant event during the development of cardiomyopathy. *let-7* has been shown to target TGF-3β and regulate cardiomyocyte apoptosis after MI [149]. Bioinformatic predictions have shown several genes, such as TBX5, FOXP1, HAND1, AKT2, and PPARGC1A, which are related to cardiac development, to be targets of different *let-7s* (*let-7a/7d/7e/7f*) [150]. These findings suggest that *let-7* might contribute to heart development and/or heart diseases, potentially as a target for cardiovascular disease therapeutics [36]. A recent study showed that accumulation of hnRNPA1 and TDP-43 are associated with neurodegenerative disease and ischemic stroke [151,152]. Another study has shown that TRAIL-R2 is one of the most powerful biomarkers for predicting long-term mortality in many diseases, such as diabetes, heart failure, myocardial infarction, smoking, and hypercholesterolemia [153]. MCPIP1 has recently been shown to negatively regulate inflammatory responses after ischemic stroke, to enhance blood–brain barrier integrity, and to be neuroprotective [154]. Additionally, RBM3 has recently been shown to be neuroprotective and positively correlate with good ischemic stroke outcomes [155]. *let-7* is a major player in diverse processes; any dysfunction in *let-7* regulation can cause a disease state, and it is essential to study how these regulatory elements link with inflammatory diseases. Taking all these together, since all the above-mentioned proteins are involved in *let-7* miR regulation, it is reasonable to suggest that *let-7s* play a significant role in the aforementioned outcomes of these regulators.

In recent years, advanced bioinformatic techniques allowed characterization of many circular RNAs and long noncoding RNAs. Both of these RNAs serve as competitive endogenous RNA (ceRNA) regulators for miRs. ceRNAs work as sponges and prevent miRs from acting on their target mRNA transcripts. Hundreds of ceRNAs have been described, and some of them are involved in *let-7* miRs regulation; however, their involvement in stroke or other cardiovascular diseases still remains to be explored.

## 4. Conclusions

It has been shown both in vivo and in vitro that *let-7* miRs are involved in numerous cellular processes, inflammation, immunity, and protective functions. Tissue- and condition-specific *let-7* expression is tightly regulated. The majority of the *let-7’* regulatory proteins are well studied in development, proliferation, differentiation, and cancer, but their roles in inflammation, cardiovascular disease, and/or stroke are not well studied. Further knowledge of the regulation of *let-7* is crucial for therapeutic advances.

## Figures and Tables

**Table 1:** *let-7* regulators.

Regulatory Protein	*let-7* Family	*Pri-let-7* (nucleus-Drosha) or *Pre-let-7* (cytoplasm-Dicer) or Mature *let-7* (RISC)	Promote or Suppress	Mechanism	References
**DAF-12**	*let-7* family	Transcriptional/*pri-let-7*	Promote/Suppress	Unliganded DAF-12 represses *let-7* and liganded DAF-12 promotes *let-7* transcriptionally through binding to *pri-let-7* 3′-UTR *Pri-let-7s* synthesis	[1,68,69]
**MYC**	*let-7a, 7d, 7f, 7g*	Transcriptional/*pri-let-7*	Suppress	MYC represses *let-7* at the upstream promoter region	[70,71]
**LIN42**	*let-7* family (*let-7a, 7b homologs)*	Transcriptional/*pri-let-7*	Suppress	Suppresses *let-7* transcriptionally by binding to the pri-*let-7* 3-UTR	[1,72,73]
**LIN28A-TUTases4/7**	*let-7a, 7b, 7d, 7g, 7i*	*Pri-let-7/Pre-let-7*	Suppress	Represses *let-7s* through TUTase-dependent uridylation of *pre-let7s*	[74,75,76,77,78,79,80]
**LIN28B**	*let-7a, 7d, 7f, 7g, 7i*	*Pri-let-7*	Suppress	Represses *let-7s* by sequestering *pri-let-7s* into the nucleolus	[79,81]
**TUTases2/4/7**	*let-7a, 7b, 7d, 7f, 7g, 7i, miR-98*	*Pre-let-7*	Promote	Promotes *let-7s* by mono-uridylating group II *pre-let-7s*, which enhances Dicer processing	[82]
**FHIT**	*let-7a, 7b, 7d, 7f, 7g*	*Pri-let-7*	Suppress	Induces LIN28B leading to suppression of *let-7s* through Lin28/Let-7 axis	[83,84]
**MUC1-C**	*let-7c*	*Pri-let-7*	Suppress	Translocates into the nucleus and interacts with NF-κB to activate Lin28B, leading to *let-7s* repression through Lin28/Let-7 axis	[85,86,87]
**MSI1**	*let-7b, 7g, miR-98*	*Pri-let-7*	Suppress	Can bind to target *pri-let-7s* 3′-UTR to repress transcription Recruits LIN28 to the nucleus and represses *let-7s* through Lin28/Let-7 axis	[88]
**SSB**	*let-7a, 7b, 7c, 7d, 7e, 7f, 7g, 7i*	*Pri-let-7*	Suppress	Enhances LIN28B transcription and represses *let-7s* through Lin28/Let-7 axis	[89,90,91]
**TRIM25**	*let-7a*	*Pre-let-7*	Suppress	A cofactor for Lin28A/TUTase4-mediated uridylation	[77,78,92]
**TRIM71**	*let-7a, 7b, 7c, 7d, 7e, 7f, 7g, 7i, miR-98*	*Pre-let-7/*Mature *let-7*	Promote	Negatively regulates Lin28B through polyubiquitination Degradation of Ago2	[93,94,95,96]
**TTP**	*Let-7a, 7b, 7f, 7g*	*Pre-let-7*	Promote	Downregulates LIN28A through binding to its AREs	[97,98]
**YAP**	*let-7a*	*Pri-let-7*	Suppress	YAP translocates into the nucleus and sequesters DDX17 and interferes with Drosha processing	[99,100]
**ADAR1**	*let-7a, 7d, 7e, 7f; let-7 family*	*Pri-let-7/Pre-let-7*	Promote	Enhances Drosha and Dicer processing through direct interactions	[101,102,103,104,105,106]
**hnRNPA1**	*let-7a*	*Pri-let-7*	Suppress	Direct binding to *pri-let-7* Reduces Drosha processing	[107,108,109,110]
**KSRP**	*let-7a*	*Pri-let-7/Pre-let-7*	Promote	Direct binding to *pri-let-7* and *pre-let-7* *Enhances Drosha processing*	[109,111,112]
**TDP-43**	*let-7b*	*Pri-let-7*	Promote	Interacts with *pri-let-7* Enhances Drosha processing	[109,113,114,115,116]
**TRAIL-R2**	*let-7a, 7b, 7c, 7d, 7e, 7g*	*Pri-let-7*	Suppress	Interacts with Drosha complex to reduce *pri-let-7* processing	[117,118,119]
**NF90/NF45**	*let-7a*	*Pri-let-7*	Suppress	Directly binds to *pri-let-7s* and reduces affinity Interacts with Drosha complex	[117,120]
**BRCA1/SMAD/p53/DHX9**	*let-7a*	*Pri-let-7*	Promote	Enhances *pri-let-7s* processing mediated by Drosha complex Binds *pri-let-7s*	[121,122,123,124,125,126]
**SNIP1**	*let-7i*	*Pri-let-7*	Promote	Likely binds *pri-let-7* and enhances Drosha processing	[127]
**STAUFEN**	*let-7s*	*Pri-let-7*	Suppress	Likely binds to pri-let-7 3′-UTR and alters structural integrity	[128,129]
**SYNCRIP**	*let-7a*	*Pri-let-7*	Promote	Binds to pri-let-7 terminal loop and enhances Drosha processing	[28]
**BCDIN3D**	*let-7b, 7d, 7d, 7e, 7f, 7g, 7i, miR-98*	*Pre-let-7*	Promote	Methylates *pre-let-7s* and enhances Dicer processing	[130,131]
**MCPIP1**	*let-7g*	*Pre-let-7*	Suppress	Cleaves terminal loops on the pre-let-7s leading to degradation	[131,132]
**TBM3**	*let-7a, 7g, 7i*	*Pre-let-7*	Promote	Binds *pre-let-7s*/enhance Dicer	[133,134]

## References

[1] Reinhart B.J., Slack F.J., Basson M., Pasquinelli A.E., Bettinger J.C., Rougvie A.E., Horvitz H.R., Ruvkun G. (2000). The 21-nucleotide let-7 RNA regulates developmental timing in Caenorhabditis elegans. Nat. Cell Biol..

[2] Ali A., Bouma G.J., Anthony R.V., Winger Q.A. (2020). The Role of LIN28-let-7-ARID3B Pathway in Placental Development. Int. J. Mol. Sci..

[3] Roush S., Slack F.J. (2008). The let-7 family of microRNAs. Trends Cell Biol..

[4] Hertel J., Bartschat S., Wintsche A., Otto C., Stadler P.F., The Students of the Bioinformatics Computer Lab (2012). Evolution of the let-7 microRNA Family. RNA Biol..

[5] Wu Y.-C., Chen C.-H., Mercer A., Sokol N.S. (2012). let-7-Complex MicroRNAs Regulate the Temporal Identity of Drosophila Mushroom Body Neurons via chinmo. Dev. Cell.

[6] Lee H., Han S., Kwon C.S., Lee D. (2016). Biogenesis and regulation of the let-7 miRNAs and their functional implications. Protein Cell.

[7] Bartel D.P. (2004). MicroRNAs: Genomics, Biogenesis, Mechanism, and Function. Cell.

[8] Eder P.S., Devine R.J., Dagle J., Walder J.A. (1991). Substrate Specificity and Kinetics of Degradation of Antisense Oligonucleotides by a 3′ Exonuclease in Plasma. Antisense Res. Dev..

[9] Li S., Wang X., Gu Y., Chen C., Wang Y., Liu J., Hu W., Yu B., Wang Y., Ding F. (2015). Let-7 microRNAs Regenerate Peripheral Nerve Regeneration by Targeting Nerve Growth Factor. Mol. Ther..

[10] Gérard C., Lemaigre F., Gonze D. (2019). Modeling the Dynamics of Let-7-Coupled Gene Regulatory Networks Linking Cell Proliferation to Malignant Transformation. Front. Physiol..

[11] Wei J., Li H., Wang S., Li T., Fan J., Liang X., Li J., Han Q., Zhu L., Fan L. (2014). let-7 Enhances Osteogenesis and Bone Formation While Repressing Adipogenesis of Human Stromal/Mesenchymal Stem Cells by Regulating HMGA2. Stem Cells Dev..

[12] Tolonen A., Magga J., Szabó Z., Viitala P., Gao E., Moilanen A., Ohukainen P., Vainio L., Koch W.J., Kerkelä R. (2014). Inhibition of Let-7 micro RNA attenuates myocardial remodeling and improves cardiac function postinfarction in mice. Pharmacol. Res. Perspect..

[13] Jiang X.-M., Yu X.-F., Wang Z.-K., Liu F.-F., Wang Y. (2016). Let-7a gene knockdown protects against cerebral ischemia/reperfusion injury. Neural Regen. Res..

[14] Na H.S.T., Nuo M., Meng Q.-T., Xia Z.-Y. (2019). The Pathway of Let-7a-1/2-3p and HMGB1 Mediated Dexmedetomidine Inhibiting Microglia Activation in Spinal Cord Ischemia-Reperfusion Injury Mice. J. Mol. Neurosci..

[15] Cho K.J., Song J., Oh Y., Lee J.E. (2015). MicroRNA-Let-7a regulates the function of microglia in inflammation. Mol. Cell. Neurosci..

[16] Wang S., Zhou H., Wu D., Ni H., Chen Z., Chen C., Xiang Y., Dai K., Chen X., Li X. (2019). MicroRNA let-7a regulates angiogenesis by targetingTGFBR3mRNA. J. Cell. Mol. Med..

[17] Ham O., Lee S.-Y., Lee C.Y., Park J.-H., Lee J., Seo H.-H., Cha M.-J., Choi E., Kim S., Hwang K.-C. (2015). let-7b suppresses apoptosis and autophagy of human mesenchymal stem cells transplanted into ischemia/reperfusion injured heart 7by targeting caspase-3. Stem Cell Res. Ther..

[18] Long G., Wang F., Li H., Yin Z., Sandip C., Lou Y., Wang Y., Chen C., Wang D.W. (2013). Circulating miR-30a, miR-126 and let-7b as biomarker for ischemic stroke in humans. BMC Neurol..

[19] Chi N., Chiou H., Chou S., Hu C., Chen K., Chang C., Hsieh Y. (2020). Hyperglycemia-related FAS gene and hsa-let-7b-5p as markers of poor outcomes for ischaemic stroke. Eur. J. Neurol..

[20] Li S., Chen L., Zhou X., Li J., Liu J. (2019). miRNA-223-3p and let-7b-3p as potential blood biomarkers associated with the ischemic penumbra in rats. Acta Neurobiol. Exp..

[21] Bernstein D.L., Zuluaga-Ramirez V., Gajghate S., Reichenbach N.L., Polyak B., Persidsky Y., Rom S. (2019). miR-98 reduces endothelial dysfunction by protecting blood–brain barrier (BBB) and improves neurological outcomes in mouse ischemia/reperfusion stroke model. Br. J. Pharmacol..

[22] Li H.-W., Meng Y., Xie Q., Yi W.-J., Lai X.-L., Bian Q., Wang J., Wang J.-F., Yu G. (2015). miR-98 protects endothelial cells against hypoxia/reoxygenation induced-apoptosis by targeting caspase-3. Biochem. Biophys. Res. Commun..

[23] Rom S., Dykstra H., Zuluaga-Ramirez V., Reichenbach N.L., Persidsky Y. (2015). miR-98 and let-7g* Protect the Blood-Brain Barrier Under Neuroinflammatory Conditions. Br. J. Pharmacol..

[24] Bernstein D.L., Rom S. (2020). Let-7g* and miR-98 Reduce Stroke-Induced Production of Proinflammatory Cytokines in Mouse Brain. Front. Cell Dev. Biol..

[25] Bernstein D.L., Gajghate S., Reichenbach N.L., Winfield M., Persidsky Y., Heldt N.A., Rom S. (2020). let-7g counteracts endothelial dysfunction and ameliorating neurological functions in mouse ischemia/reperfusion stroke model. Brain Behav. Immun..

[26] Xiang W., Tian C., Peng S., Zhou L., Pan S., Deng Z. (2017). Let-7i attenuates human brain microvascular endothelial cell damage in oxygen glucose deprivation model by decreasing toll-like receptor 4 expression. Biochem. Biophys. Res. Commun..

[27] Jickling G.C., Ander B.P., Shroff N., Orantia M., Stamova B., Dykstra-Aiello C., Hull H., Zhan X., Liu D., Sharp F.R. (2016). Leukocyte response is regulated by microRNA let7i in patients with acute ischemic stroke. Neurology.

[28] Chen D., Li L., Wang Y., Xu R., Peng S., Zhou L., Deng Z. (2020). Ischemia-reperfusion injury of brain induces endothelial-mesenchymal transition and vascular fibrosis via activating let-7i/TGF-βR1 double-negative feedback loop. FASEB J..

[29] Lin Z., Ge J., Wang Z., Ren J., Wang X., Xiong H., Gao J., Zhang Y., Zhang Q. (2017). Let-7e modulates the inflammatory response in vascular endothelial cells through ceRNA crosstalk. Sci. Rep..

[30] Huang S., Lv Z., Guo Y., Li L., Zhang Y., Zhou L., Yang B., Wu S., Zhang Y., Xie C. (2016). Identification of Blood Let-7e-5p as a Biomarker for Ischemic Stroke. PLoS ONE.

[31] Nguyen T., Su C., Singh M. (2018). Let-7i inhibition enhances progesterone-induced functional recovery in a mouse model of ischemia. Proc. Natl. Acad. Sci. USA.

[32] Yuan H., Zhang H., Hong L., Zhao H., Wang J., Li H., Che H., Zhang Z. (2018). MicroRNA let-7c-5p Suppressed Lipopolysaccharide-Induced Dental Pulp Inflammation by Inhibiting Dentin Matrix Protein-1-Mediated Nuclear Factor kappa B (NF-κB) Pathway In Vitro and In Vivo. Med Sci. Monit..

[33] Wong L.L., Saw E.L., Lim J.Y., Zhou Y., Richards A.M., Wang P. (2019). MicroRNA Let-7d-3p Contributes to Cardiac Protection via Targeting HMGA2. Int. J. Mol. Sci..

[34] Hsu P.-Y., Hsi E., Wang T.-M., Lin R.-T., Liao Y.-C., Juo S.-H.H. (2016). MicroRNA let-7g possesses a therapeutic potential for peripheral artery disease. J. Cell. Mol. Med..

[35] Zhuang Y., Peng H., Mastej V., Chen W. (2016). MicroRNA Regulation of Endothelial Junction Proteins and Clinical Consequence. Mediat. Inflamm..

[36] Bao M.-H., Feng X., Zhang Y.-W., Lou X.-Y., Cheng Y., Zhou H.-H. (2013). Let-7 in Cardiovascular Diseases, Heart Development and Cardiovascular Differentiation from Stem Cells. Int. J. Mol. Sci..

[37] Joshi S., Wei J., Bishopric N.H. (2016). A cardiac myocyte-restricted Lin28/let-7 regulatory axis promotes hypoxia-mediated apoptosis by inducing the AKT signaling suppressor PIK3IP1. Biochim. Biophys. Acta (BBA) Mol. Basis Dis..

[38] Hennchen M., Stubbusch J., Makhfi I.A.-E., Kramer M., Deller T., Pierre-Eugene C., Janoueix-Lerosey I., Delattre O., Ernsberger U., Schulte J.H. (2015). Lin28B and Let-7 in the Control of Sympathetic Neurogenesis and Neuroblastoma Development. J. Neurosci..

[39] Gulman N.K., Armon L., Shalit T., Urbach A. (2019). Heterochronic regulation of lung development via the Lin28-Let-7 pathway. FASEB J..

[40] Fairchild C.L.A., Cheema S.K., Wong J., Hino K., Simó S., La Torre A. (2019). Let-7 regulates cell cycle dynamics in the developing cerebral cortex and retina. Sci. Rep..

[41] Morgado A.L., Rodrigues C.M.P., Solá S. (2016). MicroRNA-145 Regulates Neural Stem Cell Differentiation Through the Sox2-Lin28/let-7 Signaling Pathway. STEM CELLS.

[42] Deng Z., Wei Y., Yao Y., Gao S., Wang X. (2020). Let-7f promotes the differentiation of neural stem cells in rats. Am. J. Transl. Res..

[43] Chen P.-Y., Qin L., Barnes C., Charisse K., Yi T., Zhang X., Ali R., Medina P.P., Yu J., Slack F.J. (2012). FGF Regulates TGF-β Signaling and Endothelial-to-Mesenchymal Transition via Control of let-7 miRNA Expression. Cell Rep..

[44] Kalomoiris S., Cicchetto A.C., Lakatos K., Nolta J.A., Fierro F.A. (2016). Fibroblast Growth Factor 2 Regulates High Mobility Group A2 Expression in Human Bone Marrow-Derived Mesenchymal Stem Cells. J. Cell. Biochem..

[45] Liao Y.-C., Wang Y.-S., Guo Y.-C., Lin W.-L., Chang M.-H., Juo S.-H.H. (2014). Let-7g Improves Multiple Endothelial Functions Through Targeting Transforming Growth Factor-Beta and SIRT-1 Signaling. J. Am. Coll. Cardiol..

[46] Mozos I., Malainer C., Horbańczuk J., Gug C., Stoian D., Luca C.T., Atanasov A.G. (2017). Inflammatory Markers for Arterial Stiffness in Cardiovascular Diseases. Front. Immunol..

[47] Tonini T., Rossi F., Claudio P.P. (2003). Molecular basis of angiogenesis and cancer. Oncogene.

[48] Isanejad A., Alizadeh A.M., Shalamzari S.A., Khodayari H., Khodayari S., Khori V., Khojastehnjad N. (2016). MicroRNA-206, let-7a and microRNA-21 pathways involved in the anti-angiogenesis effects of the interval exercise training and hormone therapy in breast cancer. Life Sci..

[49] Wang T., Wang G., Hao D., Liu X., Wang D., Ning N., Li X. (2015). Aberrant regulation of the LIN28A/LIN28B and let-7 loop in human malignant tumors and its effects on the hallmarks of cancer. Mol. Cancer.

[50] Buonfiglioli A., Efe I.E., Guneykaya D., Ivanov A., Huang Y., Orlowski E., Krüger C., Deisz R.A., Markovic D., Flüh C. (2019). let-7 MicroRNAs Regulate Microglial Function and Suppress Glioma Growth through Toll-Like Receptor 7. Cell Rep..

[51] Wu T., Jia J., Xiong X., He H., Bu L., Zhao Z., Huang C., Zhang W. (2013). Increased Expression of Lin28B Associates with Poor Prognosis in Patients with Oral Squamous Cell Carcinoma. PLoS ONE.

[52] Tristán-Ramos P., Rubio-Roldan A., Peris G., Sánchez L., Amador-Cubero S., Viollet S., Cristofari G., Heras S.R. (2020). The tumor suppressor microRNA let-7 inhibits human LINE-1 retrotransposition. Nat. Commun..

[53] Chen Y., Xie C., Zheng X., Nie X., Wang Z., Liu H., Zhao Y. (2019). LIN28/let-7/PD-L1 Pathway as a Target for Cancer Immunotherapy. Cancer Immunol. Res..

[54] Mardani R., Abadi M.H.J.N., Motieian M., Taghizadeh-Boroujeni S., Bayat A., Farsinezhad A., Gheibi-Hayat S.M., Motieian M., Pourghadamyari H. (2019). MicroRNA in leukemia: Tumor suppressors and oncogenes with prognostic potential. J. Cell. Physiol..

[55] Brennan E., Wang B., McClelland A., Mohan M., Marai M., Beuscart O., Derouiche S., Gray S., Pickering R., Tikellis C. (2017). Protective Effect of let-7 miRNA Family in Regulating Inflammation in Diabetes-Associated Atherosclerosis. Diabetes.

[56] Baer C., Squadrito M.L., Laoui D., Thompson D., Hansen S.K., Kiialainen A., Hoves S., Ries C.H., Ooi C.-H., De Palma M. (2016). Suppression of microRNA activity amplifies IFN-γ-induced macrophage activation and promotes anti-tumour immunity. Nat. Cell Biol..

[57] Li X.-X., Di X., Cong S., Wang Y., Wang K. (2018). The role of let-7 and HMGA2 in the occurrence and development of lung cancer: A systematic review and meta-analysis. Eur. Rev. Med. Pharmacol. Sci..

[58] Tsang W.P., Kwok T.T. (2008). Let-7a microRNA suppresses therapeutics-induced cancer cell death by targeting caspase-3. Apoptosis.

[59] Zha W., Guan S., Liu N., Li Y., Tian Y., Chen Y., Wang Y., Wu F. (2020). Let-7a inhibits Bcl-xl and YAP1 expression to induce apoptosis of trophoblast cells in early-onset severe preeclampsia. Sci. Total Environ..

[60] Zhang H., Xiong X., Gu L., Xie W., Zhao H. (2018). CD4 T cell deficiency attenuates ischemic stroke, inhibits oxidative stress, and enhances Akt/mTOR survival signaling pathways in mice. Chin. Neurosurg. J..

[61] Wang G., Zhang Z., Ayala C., Dunet D.O., Fang J., George M.G. (2014). Costs of Hospitalization for Stroke Patients Aged 18-64 Years in the United States. J. Stroke Cerebrovasc. Dis..

[62] Shi X.-Y., Wang H., Wang W., Gu Y.-H. (2020). MiR-98-5p regulates proliferation and metastasis of MCF-7 breast cancer cells by targeting Gab2. Eur. Rev. Med. Pharmacol. Sci..

[63] Chen Y.-L., Qiao Y.-C., Xu Y., Ling W., Pan Y.-H., Huang Y.-C., Geng L.-J., Zhao H.-L., Zhang X.-X. (2017). Serum TNF-α concentrations in type 2 diabetes mellitus patients and diabetic nephropathy patients: A systematic review and meta-analysis. Immunol. Lett..

[64] Hu C., Huang S., Wu F., Ding H. (2018). miR-98 inhibits cell proliferation and induces cell apoptosis by targeting MAPK6 in HUVECs. Exp. Ther. Med..

[65] Zhao C., Popel A.S. (2015). Computational Model of MicroRNA Control of HIF-VEGF Pathway: Insights into the Pathophysiology of Ischemic Vascular Disease and Cancer. PLoS Comput. Biol..

[66] Wang L., Lin Z.Q., Wong A. (2020). COVID-Net: A tailored deep convolutional neural network design for detection of COVID-19 cases from chest X-ray images. Sci. Rep..

[67] Chirshev E., Oberg K., Ioffe Y.J., Unternaehrer J.J. (2019). Let-7as biomarker, prognostic indicator, and therapy for precision medicine in cancer. Clin. Transl. Med..

[68] Hammell C.M., Karp X., Ambros V. (2009). A feedback circuit involving let-7-family miRNAs and DAF-12 integrates environmental signals and developmental timing in Caenorhabditis elegans. Proc. Natl. Acad. Sci. USA.

[69] Bethke A., Fielenbach N., Wang Z., Mangelsdorf D.J., Antebi A. (2009). Nuclear Hormone Receptor Regulation of MicroRNAs Controls Developmental Progression. Science.

[70] Chang T.-C., Yu D., Lee Y.-S., Wentzel E.A., Arking D.E., West K.M., Dang C.V., Thomas-Tikhonenko A., Mendell J.T. (2007). Widespread microRNA repression by Myc contributes to tumorigenesis. Nat. Genet..

[71] Wang Z., Lin S., Li J.J., Xu Z., Yao H., Zhu X., Xie D., Shen Z., Sze J., Li K. (2011). MYC Protein Inhibits Transcription of the MicroRNA Cluster MC-let-7a-1∼let-7d via Noncanonical E-box*. J. Biol. Chem..

[72] Van Wynsberghe P.M., Finnegan E.F., Stark T., Angelus E.P., Homan K.E., Yeo E., Pasquinelli A.E. (2014). The Period protein homolog LIN-42 negatively regulates microRNA biogenesis in C. elegans. Dev. Biol..

[73] McCulloch K.A., Rougvie A.E. (2014). Caenorhabditis elegans period homolog lin-42 regulates the timing of heterochronic miRNA expression. Proc. Natl. Acad. Sci. USA.

[74] Heo I., Joo C., Cho J., Ha M., Han J., Kim V.N. (2008). Lin28 Mediates the Terminal Uridylation of let-7 Precursor MicroRNA. Mol. Cell.

[75] Rybak A., Fuchs H., Smirnova L., Brandt C., Pohl E.E., Nitsch R., Wulczyn F.G. (2008). A feedback loop comprising lin-28 and let-7 controls pre-let-7 maturation during neural stem-cell commitment. Nat. Cell Biol..

[76] Viswanathan S., Daley G.Q., Gregory R.I. (2008). Selective Blockade of MicroRNA Processing by Lin28. Science.

[77] Hagan J.P., Piskounova E., Gregory R.I. (2009). Lin28 recruits the TUTase Zcchc11 to inhibit let-7 maturation in mouse embryonic stem cells. Nat. Struct. Mol. Biol..

[78] Heo I., Joo C., Kim Y.-K., Ha M., Yoon M.-J., Cho J., Yeom K.-H., Han J., Kim V.N. (2009). TUT4 in Concert with Lin28 Suppresses MicroRNA Biogenesis through Pre-MicroRNA Uridylation. Cell.

[79] Piskounova E., Polytarchou C., Thornton J.E., Lapierre R.J., Pothoulakis C., Hagan J.P., Iliopoulos D., Gregory R.I. (2011). Lin28A and lin28B Inhibit let-7 microRNA biogenesis by distinct mechanisms. Cell.

[80] Thornton J.E., Chang H.-M., Piskounova E., Gregory R.I. (2012). Lin28-mediated control of let-7 microRNA expression by alternative TUTases Zcchc11 (TUT4) and Zcchc6 (TUT7). RNA.

[81] Newman M.A., Thomson J.M., Hammond S.M. (2008). Lin-28 interaction with the Let-7 precursor loop mediates regulated microRNA processing. RNA.

[82] Heo I., Ha M., Lim J., Yoon M.-J., Park J.-E., Kwon S.C., Chang H., Kim V.N. (2012). Mono-Uridylation of Pre-MicroRNA as a Key Step in the Biogenesis of Group II let-7 MicroRNAs. Cell.

[83] Barnes L.D., Garrison P.N., Siprashvili Z., Guranowski A., Robinson A.K., Ingram S.W., Croce C.M., Ohta M., Huebner K. (1996). Fhit, a Putative Tumor Suppressor in Humans, Is a Dinucleoside 5‘,5‘ ‘‘-P1,P3-Triphosphate Hydrolase†. Biochemistry.

[84] Chae H.-J., Seo J.B., Kim S.-H., Jeon Y.-J., Suh S.-S. (2021). Fhit induces the reciprocal suppressions between Lin28/Let-7 and miR-17/92miR. Int. J. Med Sci..

[85] Kufe D.W. (2009). Mucins in cancer: Function, prognosis and therapy. Nat. Rev. Cancer.

[86] Alam M., Ahmad R., Rajabi H., Kufe D. (2015). MUC1-C Induces the LIN28B→LET-7→HMGA2 Axis to Regulate Self-Renewal in NSCLC. Mol. Cancer Res..

[87] Kufe D.W. (2012). MUC1-C oncoprotein as a target in breast cancer: Activation of signaling pathways and therapeutic approaches. Oncogene.

[88] Kawahara H., Okada Y., Imai T., Iwanami A., Mischel P.S., Okano H. (2011). Musashi1 Cooperates in Abnormal Cell Lineage Protein 28 (Lin28)-mediated Let-7 Family MicroRNA Biogenesis in Early Neural Differentiation. J. Biol. Chem..

[89] Kim S.H., Park B.-O., Kim K., Park B.C., Park S.G., Kim J.-H., Kim S. (2021). Sjögren Syndrome antigen B regulates LIN28-let-7 axis in Caenorhabditis elegans and human. Biochim. Biophys. Acta (BBA) Bioenerg..

[90] Teplova M., Yuan Y.-R., Phan A.T., Malinina L., Ilin S., Teplov A., Patel D.J. (2006). Structural Basis for Recognition and Sequestration of UUUOH 3′ Temini of Nascent RNA Polymerase III Transcripts by La, a Rheumatic Disease Autoantigen. Mol. Cell.

[91] Stefano J.E. (1984). Purified lupus antigen la recognizes an oligouridylate stretch common to the 3′ termini of RNA polymerase III transcripts. Cell.

[92] Choudhury N.R., Nowak J.S., Zuo J., Rappsilber J., Spoel S.H., Michlewski G. (2014). Trim25 Is an RNA-Specific Activator of Lin28a/TuT4-Mediated Uridylation. Cell Rep..

[93] Lee S.H., Cho S., Kim M.S., Choi K., Cho J.Y., Gwak H.-S., Kim Y.-J., Yoo H., Lee S.-H., Park J.B. (2014). The ubiquitin ligase human TRIM71 regulates let-7 microRNA biogenesis via modulation of Lin28B protein. Biochim. Biophys. Acta (BBA) Bioenerg..

[94] Chang H.-M., Martinez N.J., Thornton J.E., Hagan J.P., Nguyen K.D., Gregory R.I. (2012). Trim71 cooperates with microRNAs to repress Cdkn1a expression and promote embryonic stem cell proliferation. Nat. Commun..

[95] Rybak A., Fuchs H., Hadian K., Smirnova L., Wulczyn E.A., Michel G., Nitsch R., Krappmann D., Wulczyn F.G. (2009). The let-7 target gene mouse lin-41 is a stem cell specific E3 ubiquitin ligase for the miRNA pathway protein Ago2. Nat. Cell Biol..

[96] Liu Q., Chen X., Novak M.K., Zhang S., Hu W. (2021). Repressing Ago2 mRNA translation by Trim71 maintains pluripotency through inhibiting let-7 microRNAs. eLife.

[97] Kim C.W., Vo M.-T., Kim H.K., Lee H.H., Yoon N.A., Lee B.J., Min Y.J., Joo W.D., Cha H.J., Park J.W. (2011). Ectopic over-expression of tristetraprolin in human cancer cells promotes biogenesis of let-7 by down-regulation of Lin28. Nucleic Acids Res..

[98] Shaw G., Kamen R. (1986). A conserved AU sequence from the 3′ untranslated region of GM-CSF mRNA mediates selective mRNA degradation. Cell.

[99] Mori M., Triboulet R., Mohseni M., Schlegelmilch K., Shrestha K., Camargo F.D., Gregory R.I. (2014). Hippo Signaling Regulates Microprocessor and Links Cell-Density-Dependent miRNA Biogenesis to Cancer. Cell.

[100] Chaulk S.G., Lattanzi V.J., Hiemer S.E., Fahlman R.P., Varelas X. (2014). The Hippo Pathway Effectors TAZ/YAP Regulate Dicer Expression and MicroRNA Biogenesis through Let-7. J. Biol. Chem..

[101] Chawla G., Sokol N.S. (2014). ADAR mediates differential expression of polycistronic microRNAs. Nucleic Acids Res..

[102] Bahn J.H., Ahn J., Lin X., Zhang Q., Lee J.-H., Civelek M., Xiao X. (2015). Genomic analysis of ADAR1 binding and its involvement in multiple RNA processing pathways. Nat. Commun..

[103] Nemlich Y., Greenberg E., Ortenberg R., Besser M.J., Barshack I., Jacob-Hirsch J., Jacoby E., Eyal E., Rivkin L., Prieto V.G. (2013). MicroRNA-mediated loss of ADAR1 in metastatic melanoma promotes tumor growth. J. Clin. Investig..

[104] Zipeto M.A., Court A.C., Sadarangani A., Santos N.P.D., Balaian L., Chun H.-J., Pineda G., Morris S.R., Mason C.N., Geron I. (2016). ADAR1 Activation Drives Leukemia Stem Cell Self-Renewal by Impairing Let-7 Biogenesis. Cell Stem Cell.

[105] Ota H., Sakurai M., Gupta R., Valente L., Wulff B.-E., Ariyoshi K., Iizasa H., Davuluri R.V., Nishikura K. (2013). ADAR1 Forms a Complex with Dicer to Promote MicroRNA Processing and RNA-Induced Gene Silencing. Cell.

[106] Germanguz I., Lowry W.E. (2016). RNA editing as an activator of self-renewal in cancer. Stem Cell Investig..

[107] Michlewski G., Guil S., Semple C., Cáceres J.F. (2008). Posttranscriptional Regulation of miRNAs Harboring Conserved Terminal Loops. Mol. Cell.

[108] Jain N., Lin H.-C., Morgan C.E., Harris M.E., Tolbert B.S. (2017). Rules of RNA specificity of hnRNP A1 revealed by global and quantitative analysis of its affinity distribution. Proc. Natl. Acad. Sci. USA.

[109] Michlewski G., Cáceres J.F. (2010). Antagonistic role of hnRNP A1 and KSRP in the regulation of let-7a biogenesis. Nat. Struct. Mol. Biol..

[110] Burd C., Dreyfuss G. (1994). RNA binding specificity of hnRNP A1: Significance of hnRNP A1 high-affinity binding sites in pre-mRNA splicing. EMBO J..

[111] Trabucchi M., Briata P., Garcia-Mayoral M., Haase A.D., Filipowicz W., Ramos A., Gherzi R., Rosenfeld M.G. (2009). The RNA-binding protein KSRP promotes the biogenesis of a subset of microRNAs. Nat. Cell Biol..

[112] Gherzi R., Chen C.-Y., Ramos A., Briata P. (2014). KSRP Controls Pleiotropic Cellular Functions. Semin. Cell Dev. Biol..

[113] Guil S., Caceres J. (2007). The multifunctional RNA-binding protein hnRNP A1 is required for processing of miR-18a. Nat. Struct. Mol. Biol..

[114] Buratti E., Baralle F.E. (2008). Multiple roles of TDP-43 in gene expression, splicing regulation, and human disease. Front. Biosci..

[115] Buratti E., De Conti L., Stuani C., Romano M., Baralle M., Baralle F. (2010). Nuclear factor TDP-43 can affect selected microRNA levels. FEBS J..

[116] Kawahara Y., Mieda-Sato A. (2012). TDP-43 promotes microRNA biogenesis as a component of the Drosha and Dicer complexes. Proc. Natl. Acad. Sci. USA.

[117] Haselmann V., Kurz A., Bertsch U., Hübner S., Olempska–Müller M., Fritsch J., Häsler R., Pickl A., Fritsche H., Annewanter F. (2014). Nuclear Death Receptor TRAIL-R2 Inhibits Maturation of Let-7 and Promotes Proliferation of Pancreatic and Other Tumor Cells. Gastroenterology.

[118] Boyerinas B., Park S.-M., Hau A., Murmann A.E., Peter M.E. (2010). The role of let-7 in cell differentiation and cancer. Endocr.-Relat. Cancer.

[119] Salzman D.W., Shubert-Coleman J., Furneaux H. (2007). P68 RNA Helicase Unwinds the Human let-7 MicroRNA Precursor Duplex and Is Required for let-7-directed Silencing of Gene Expression. J. Biol. Chem..

[120] Sakamoto S., Aoki K., Higuchi T., Todaka H., Morisawa K., Tamaki N., Hatano E., Fukushima A., Taniguchi T., Agata Y. (2009). The NF90-NF45 Complex Functions as a Negative Regulator in the MicroRNA Processing Pathway. Mol. Cell. Biol..

[121] Kawai S., Amano A. (2012). BRCA1 regulates microRNA biogenesis via the DROSHA microprocessor complex. J. Cell Biol..

[122] Anderson S.F., Schlegel B.P., Nakajima T., Wolpin E.S., Parvin J.D. (1998). BRCA1 protein is linked to the RNA polymerase II holoenzyme complex via RNA helicase A. Nat. Genet..

[123] Wilson B.J., Giguère V. (2007). Identification of novel pathway partners of p68 and p72 RNA helicases through Oncomine meta-analysis. BMC Genom..

[124] Fuller-Pace F.V. (2006). DExD/H box RNA helicases: Multifunctional proteins with important roles in transcriptional regulation. Nucleic Acids Res..

[125] Davis B.N., Hilyard A.C., Lagna G., Hata A. (2008). SMAD proteins control DROSHA-mediated microRNA maturation. Nat. Cell Biol..

[126] Dubrovska A., Kanamoto T., Lomnytska M., Heldin C.-H., Volodko N., Souchelnytskyi S. (2005). TGFβ1/Smad3 counteracts BRCA1-dependent repair of DNA damage. Oncogene.

[127] Yu B., Bi L., Zheng B., Ji L., Chevalier D., Agarwal M., Ramachandran V., Li W., Lagrange T., Walker J.C. (2008). The FHA domain proteins DAWDLE in Arabidopsis and SNIP1 in humans act in small RNA biogenesis. Proc. Natl. Acad. Sci. USA.

[128] Li P., Yang X., Wasser M., Cai Y., Chia W. (1997). Inscuteable and Staufen Mediate Asymmetric Localization and Segregation of prospero RNA during Drosophila Neuroblast Cell Divisions. Cell.

[129] Ren Z., Veksler-Lublinsky I., Morrissey D., Ambros V. (2016). Staufen Negatively Modulates MicroRNA Activity in Caenorhabditis elegans. G3 Genes|Genomes|Genet..

[130] Reinsborough C.W., Ipas H., Abell N.S., Gouws E.B., Williams J.P., Mercado M., Berg C.V.D., Xhemalçe B. (2021). BCDIN3D RNA methyltransferase stimulates Aldolase C expression and glycolysis through let-7 microRNA in breast cancer cells. Oncogene.

[131] Xhemalce B., Robson S.C., Kouzarides T. (2012). Human RNA Methyltransferase BCDIN3D Regulates MicroRNA Processing. Cell.

[132] Suzuki H., Arase M., Matsuyama H., Choi Y.L., Ueno T., Mano H., Sugimoto K., Miyazono K. (2011). MCPIP1 Ribonuclease Antagonizes Dicer and Terminates MicroRNA Biogenesis through Precursor MicroRNA Degradation. Mol. Cell.

[133] Pilotte J., Dupont-Versteegden E.E., Vanderklish P.W. (2011). Widespread Regulation of miRNA Biogenesis at the Dicer Step by the Cold-Inducible RNA-Binding Protein, RBM3. PLoS ONE.

[134] Dannoab S., Nishiyamaa H., Higashitsujia H., Yokoia H., Xuea J.-H., Itoha K., Matsudab T., Fujita J. (1997). Increased Transcript Level of RBM3, a Member of the Glycine-Rich RNA-Binding Protein Family, in Human Cells in Response to Cold Stress. Biochem. Biophys. Res. Commun..

[135] Großhans H., Johnson T., Reinert K.L., Gerstein M., Slack F.J. (2005). The Temporal Patterning MicroRNA let-7 Regulates Several Transcription Factors at the Larval to Adult Transition in C. elegans. Dev. Cell.

[136] Kumar M.S., Lu J., Mercer K.L., Golub T.R., Jacks T. (2007). Impaired microRNA processing enhances cellular transformation and tumorigenesis. Nat. Genet..

[137] Nguyen D.T.T., Richter D., Michel G., Mitschka S., Kolanus W., Cuevas E., Wulczyn F.G. (2017). The ubiquitin ligase LIN41/TRIM71 targets p53 to antagonize cell death and differentiation pathways during stem cell differentiation. Cell Death Differ..

[138] Mooijaart S., Brandt B., Baldal E., Pijpe J., Kuningas M., Beekman M., Zwaan B., Slagboom P., Westendorp R., Van Heemst D. (2005). *C. elegans* DAF-12, Nuclear Hormone Receptors and human longevity and disease at old age. Ageing Res. Rev..

[139] Balzer E., Moss E.G. (2007). Localization of the Developmental Timing Regulator Lin28 to mRNP Complexes, P-bodies and Stress Granules. RNA Biol..

[140] Carballo E., Lai W.S., Blackshear P.J. (1998). Feedback Inhibition of Macrophage Tumor Necrosis Factor-α Production by Tristetraprolin. Science.

[141] Lykke-Andersen J. (2005). Recruitment and activation of mRNA decay enzymes by two ARE-mediated decay activation domains in the proteins TTP and BRF-1. Genes Dev..

[142] Yang W., Chendrimada T.P., Wang Q., Higuchi M., Seeburg P.H., Shiekhattar R., Nishikura K. (2005). Modulation of microRNA processing and expression through RNA editing by ADAR deaminases. Nat. Struct. Mol. Biol..

[143] Suzuki H., Yamagata K., Sugimoto K., Iwamoto T., Kato S., Miyazono K. (2009). Modulation of microRNA processing by p53. Nat. Cell Biol..

[144] Chen Y., Chan J., Chen W., Li J., Sun M., Kannan G.S., Mok Y.-K., Yuan Y.A., Jobichen C. (2020). SYNCRIP, a new player in pri-let-7a processing. RNA.

[145] Kim I.-M., Wang Y., Park K.-M., Tang Y., Teoh J.-P., Vinson J., Traynham C.J., Pironti G., Mao L., Su H. (2014). β-Arrestin1–Biased β 1 -Adrenergic Receptor Signaling Regulates MicroRNA Processing. Circ. Res..

[146] Li X., Lin W.-J., Chen C.-Y., Si Y., Zhang X., Lu L., Suswam E., Zheng L., King P.H. (2012). KSRP: A checkpoint for inflammatory cytokine production in astrocytes. Glia.

[147] Winzen R., Thakur B.K., Dittrich-Breiholz O., Shah M., Redich N., Dhamija S., Kracht M., Holtmann H. (2007). Functional Analysis of KSRP Interaction with the AU-Rich Element of Interleukin-8 and Identification of Inflammatory mRNA Targets. Mol. Cell. Biol..

[148] Zhu H., Gui Q., Hui X., Wang X., Jiang J., Ding L., Sun X., Wang Y., Chen H. (2017). TGF-β1/Smad3 Signaling Pathway Suppresses Cell Apoptosis in Cerebral Ischemic Stroke Rats. Med. Sci. Monit..

[149] Chen C.-Y., Choong O.K., Liu L.-W., Cheng Y.-C., Li S.-C., Yen C.Y., Wu M.-R., Chiang M.-H., Tsang T.-J., Wu Y.-W. (2019). MicroRNA let-7-TGFBR3 signalling regulates cardiomyocyte apoptosis after infarction. EBioMedicine.

[150] Cao L., Kong L.-P., Yu Z.-B., Han S.-P., Bai Y.-F., Zhu J., Hu X., Zhu C., Zhu S., Guo X.-R. (2012). microRNA expression profiling of the developing mouse heart. Int. J. Mol. Med..

[151] Kahl A., Blanco I., Jackman K., Baskar J., Milaganur Mohan H., Rodney-Sandy R., Zhang S., Iadecola C., Hochrainer K. (2018). Cerebral ischemia induces the aggregation of proteins linked to neurodegenerative diseases. Sci. Rep..

[152] Thammisetty S.S., Pedragosa J., Weng Y.C., Calon F., Planas A., Kriz J. (2018). Age-related deregulation of TDP-43 after stroke enhances NF-κB-mediated inflammation and neuronal damage. J. Neuroinflammation.

[153] Skau E., Henriksen E., Wagner P., Hedberg P., Siegbahn A., Leppert J. (2017). GDF-15 and TRAIL-R2 are powerful predictors of long-term mortality in patients with acute myocardial infarction. Eur. J. Prev. Cardiol..

[154] Jin Z., Liang J., Li J., Kolattukudy P.E. (2019). Absence of MCP-induced Protein 1 Enhances Blood–Brain Barrier Breakdown after Experimental Stroke in Mice. Int. J. Mol. Sci..

[155] Ávila-Gómez P., Vieites-Prado A., Dopico-López A., Bashir S., Fernández-Susavila H., Gubern C., Pérez-Mato M., Correa-Paz C., Iglesias-Rey R., Sobrino T. (2020). Cold stress protein RBM3 responds to hypothermia and is associated with good stroke outcome. Brain Commun..

